# A survey of chiropractors practicing in Germany: practice characteristics, professional reading habits, and attitudes and perceptions toward research

**DOI:** 10.1186/1746-1340-15-6

**Published:** 2007-05-04

**Authors:** Ilke Schwarz, Maria A Hondras

**Affiliations:** 1 Private Practice, Frankfurt, Germany; 2 Palmer Center for Chiropractic Research, Davenport, IA, USA

## Abstract

**Background:**

In 2004, a survey conducted by the European Chiropractor's Union among member countries reported that "there appears to be little interest in research among chiropractors in Germany." However, no research has tested this statement. The objective of this study was to explore the attitudes and perceptions of practicing chiropractors in Germany regarding research, to look at their reading and research habits, and to gather demographic and practice data.

**Methods:**

A questionnaire was developed and distributed among participants at a seminar held by the German Chiropractors' Association in 2005. The questionnaire was mailed to any members of the association who did not attend the seminar.

**Results:**

A total of 49 (72%) of 68 distributed questionnaires were returned. Forty-five (92%) respondents stated they would support research efforts in Germany and 15 (31%) declared interest in participating in practiced based research. An average of three hours per week were reportedly spent reading scientific literature by 44 (85%) respondents. However, few journals listed by respondents were peer-reviewed and indexed; most were newsletters of chiropractic organizations or free publications. Most participants agreed on the importance of research for the profession, but when asked about the most pressing issue for chiropractic in Germany, legislation and recognition of the profession were the dominant themes.

**Conclusion:**

The results of this survey show that there is a general interest in supporting and participating in research activities among chiropractors practicing in Germany. Next steps could consist of educating practitioners about the resources available to read and interpret the scientific literature and thus further the understanding of research.

## Background

In 2004, a survey conducted by the European Chiropractor's Union among its member countries reported that "there appears to be little interest in research among chiropractors in Germany," [1] however there were no data to support this statement. Although there is no evidence in the literature for research conducted by German chiropractors, the interest and willingness to support research in Germany have not been investigated.

The situation for chiropractors in Germany is, like in most European countries, unique. [2] Chiropractic is not regulated as a profession; instead it is grouped with other alternative therapies practiced by "lay practitioners" which in German are referred to as "Heilpraktiker". In contrast to graduates of accredited chiropractic institutions which are regulated worldwide by the Council on Chiropractic Education (CCE), there are no educational requirements for these practitioners except for an examination based on a law from 1939. [3] "Heilpraktiker" thus can perform manipulation without having proof of any type of education. American chiropractors have taken advantage of this lack of regulation and are teaching chiropractic techniques to lay practitioners. [4-6] On the other hand, the medical profession is claiming "chirotherapy" as their privilege, a qualification which can be earned by MD's after attending 320 hours of continuing education seminars. [7]

No studies have examined differences in the quality of the education or the care delivered by the three different groups of practitioners in Germany. One retrospective study looking at vertebral artery dissections after chiropractic manipulation/chirotherapy in the cervical region reported that 18 of the 36 patients were treated by orthopedic surgeons, and four were treated by chiropractors. [8] Unfortunately, the qualifications of the individual practitioners were not described in the paper, but to our knowledge there were no chiropractors who graduated from a CCE-accredited program who performed one of the reported chiropractic treatments. With over 10,000 "lay practitioners" (who may or may not perform manipulations) and several thousand medical doctors performing spinal manipulations [9], the approximately 70 chiropractors in the country who graduated from CCE accredited institutions struggle with professional identity and believe the public deserves to know the professional training of manual therapy practitioners to make informed decisions about their care. Chiropractors in Germany are also struggling to change legislation in their favor. [2]

Surveys conducted in Europe have primarily examined practitioner and patient characteristics. [10-12] In Germany, two surveys were conducted as theses by students from the Anglo-European College of Chiropractic. In 1997, Hafer investigated practitioner characteristics. [9] Patient characteristics were examined as a follow-up. (personal communication) These theses have not been published in the open literature, thus it was important to gather demographic data with this survey.

The objective of this survey was to explore the attitudes and perceptions of practicing chiropractors in Germany regarding research, to look at their reading and research habits, and to gather basic demographic and practice characteristics data.

## Methods

A self-report questionnaire was developed for this project. The target population was comprised of chiropractors practicing in Germany who graduated from an accredited chiropractic program, most of whom are members of the German Chiropractors' Association (GCA). To ascertain the number of this target population, we contacted the GCA. At the time the survey was administered (November 2005), there were 63 members actively practicing in Germany.

Two data collection methods were used for this study: face-to-face administration and a mailed questionnaire. One of the authors (IS) distributed the questionnaire to participants attending a pediatric seminar held by the GCA in early November 2005. In addition, the questionnaire was mailed in late November to members of the association who did not attend the seminar. Repeat mailings were sent to non-responders in mid-December and early January, by email or by post. All questionnaires were coded for tracking purposes and no names were obtained on individual forms. To preserve confidentiality of responses for the mailed questionnaires, one of the authors (IS) prepared the survey packet and postage-paid return envelope, to be returned to the second author (MAH).

The survey included questions regarding demographics, education, population in the area of practice, patient base, techniques and modalities utilized in practice, reading and research habits, and attitudes regarding research activities by chiropractors. Several questions were adapted from a survey used in the Netherlands and the United Kingdom. [13] Two questions about attitudes toward research were used from a survey administered to practitioners and chiropractic college faculty in the United States. [14,15] The questionnaire was pre-tested by Research Fellows at the Palmer Center for Chiropractic Research in Davenport, Iowa and clinicians at the Palmer Clinic in Rock Island, Illinois. Comments and critiques were incorporated into the final version of the questionnaire. The project was reviewed and approved by the Institutional Review Board at Palmer College of Chiropractic.

Numerical data were analyzed using SPSS 13.0. Descriptive statistics were used to report the data. Continuous data were reported as mean (SD), categorical data as count (%). Responses to open-ended questions were organized using a thematic analysis. First the responses were grouped by themes by the author (IS) and another researcher familiar with qualitative analysis (JP). The responses were compiled in an Excel spreadsheet and organized around similar themes. Then consensus was used to identify the major categories reported in the results.

## Results

Surveys were administered to all 37 chiropractors who attended the GCA pediatrics seminar, including a few chiropractors practicing outside of Germany, and 31 surveys were mailed to chiropractors who did not attend the conference. A total of 49 (72%) of 68 distributed surveys were returned; 30 (81%) from face-to-face administration and 19 (61%) from the mailed survey. Table 1 lists the demographic characteristics of respondents. Eighteen (37%) of the respondents were female, and 29 (59%) were German nationals. Three of the 29 German nationals and one of the non-German chiropractors who attended the GCA seminar reported practice locations outside of Germany. There were missing data for two non-German respondents regarding practice location.

**Table 1:** Practitioner Characteristics (n = 49)

Variable	Frequency (%)*
Age [mean (SD)]	38.3 (10.6)
Sex	
Female	18 (37)
Male	31 (63)
Nationality	
German	29 (59)
Other**	20 (41)
Chiropractic School	
Anglo-European College of Chiropractic	17 (35)
Logan College of Chiropractic	2 (4)
National College of Chiropractic	4 (8)
Northwestern College of Chiropractic	2 (4)
Palmer College Davenport, Iowa	16 (33)
Other	8 (16)
Years practiced [mean (SD)]	10.5 (9.7)
Years practiced in Germany [mean (SD)] (n = 44)	8.0 (8.3)
Years practiced at present location [mean (SD)] (n = 45)	6.3 (7.5)
Type of practice after graduation	
Associate/Employee	32 (65)
Solo Practice	7 (14)
Graduate Education Program (GEP)	24 (49)
Chiropractic Group practice	4 (8)
Multi-Specialty Practice	2 (4)
Other	3 (6)
Current type of practice	
Associate/Employee	16 (33)
Solo Practice	22 (45)
Graduate Education Program (GEP)	2 (4)
Chiropractic Group practice	12 (25)
Multi-Specialty Practice	3 (6)
Other	4 (8)
Practice setting (n = 48)	
Rural (< 20,000)	9 (18)
Town (20,000 – 50,000)	5 (10)
Small City (50,000 – 100,000)	13 (27)
Avg. City (100,000 – 250,000)	7 (14)
Large City (> 250,000)	13 (27)
Other	1 (2)
Promotion activities	30 (61)
Hours practice per week	
10–20 hrs.	4 (8)
21–30 hrs.	15 (31)
31–40 hrs.	19 (39)
More than 40 hrs.	11 (22)
Hours paperwork per week [mean (SD)]	4.2 (4.2)
Hours patient care per week [mean (SD)]	30.8 (10.9)
Patients per week [mean (SD)]	88.5 (70.1)
Percentage of patients under 6 years [mean (SD)]	6.8 (6.8)
Percentage of patients over 65 years [mean (SD)]	23.0 (12.8)
Percentage of female patients [mean (SD)]	60.2 (8.6)
Days to earliest appointment [mean (SD)]	6.9 (13.7)

*Values reported in frequencies (%) unless otherwise noted.

** Other nationalities included American, Australian, British, Canadian, Danish, Norwegian, and South African.

The majority of respondents graduated from Anglo-European College of Chiropractic in Bournemouth, UK and Palmer College of Chiropractic in Davenport, Iowa, USA. Respondents had been practicing an average of 10.5 years, and an average of eight years in Germany. Most chiropractors practiced in cities rather than rural areas. Promotion activities reported by 61% of respondents included lectures/open house, newspaper articles and advertisements, and websites. The majority of respondents reported they practiced between 31 and 40 hours per week, with a mean patient load of 89 patients per week, and an average of 60% female patients (Table 1). No demographic data are available for non-respondents.

When asked "Please list the chiropractic techniques or systems you use in your office. List in order, starting with the technique you use **most often**", [see Additional file 1], item 24, Diversified, SOT, and Gonstead techniques were listed *first *in these lists, 29, 9, and 5 times, respectively. Table 2 shows the number of times any technique was mentioned in response to item 24 in our survey. Other interventions commonly used were rehabilitation exercise, patient education, and nutrition. Low back pain (n = 42), neck pain (n = 28), and headache (n = 19) were reported as the most common presenting complaints. Vertigo/dizziness (n = 20), gastrointestinal complaints (n = 15), and infantile colic (n = 10) were the most common non-musculoskeletal complaints listed. Referrals were reported to come mostly from existing patients (n = 44), other health care professionals (n = 31), yellow page ads (n = 13), and lectures (n = 8).

**Table 2:** Techniques and Interventions Utilized (n = 49)

Variable	Frequency (%)
Technique	
Diversified/Full Spine	38 (78)
SOT	30 (61)
Activator	19 (39)
Gonstead	17 (35)
Thompson/Drop	17 (35)
Flexion/Distraction	5 (10)
Upper Cervical	7 (14)
Trigger Point Therapy	7 (14)
AK	5 (10)
Logan Basic	2 (4)
Toftness	2 (4)
Palmer Package	2 (4)
Other	16 (33)
Intervention	
Rehabilitation Exercises	38 (81)
Patient Education	28 (60)
Nutrition	11 (23)
Ergonomic Advice	3 (6)
Ice	3 (6)
Physical Therapy	3 (6)
Homeopathy	2 (4)
Soft Tissue Techniques	2 (4)

Data on reading and research habits as well as participant attitudes about research are presented in Table 3. Two respondents reported they had published in a scientific journal. Forty-four (85%) reported to read scientific journals for an average of three hours per week (median one hour, range 0.5 to 31 hours). Journals reportedly read by respondents included The Chiropractic Report (n = 15), Journal of Manipulative and Physiological Therapeutics (JMPT) (n = 11), and others to a lesser degree. The majority (92%) of respondents said they were willing to support research efforts in Germany, mostly by completing surveys or providing patient data (63% each). Fifteen participants (31%) indicated they were willing to participate in a practice based research network, 13 (27%) were willing to financially support research, and six (12%) were willing to help by writing or editing manuscripts.

**Table 3:** Reading and Research Habits (n = 49)

Variable	Frequency (%)*
Reported to read scientific journals (n = 48)	42 (86)
Reading hours per week [mean (SD)] (n = 44)	3 (5.0)
Ever published in a scientific journal	2 (4)
Use of electronic databases (n = 48)	
EMBASE	0 (0)
Index to Chiropractic Literature (ICL)	2 (4)
MANTIS	3 (6)
Medline (PubMed)	24 (49)
Other	3 (6)
None	18 (39)
Journals read (n = 37)	
Backspace	3 (6)
Backtalk	2 (4)
Chiropractic Journal	3 (6)
Chiropractic Report	15 (31)
Dynamic Chiropractic	4 (8)
FCER	2 (4)
JMPT	11 (22)
Manual Medicine	2 (4)
Manual Therapies	2 (4)
Manuelle Medizin	4 (8)
Today's Chiropractic	2 (4)
Other	19 (39)
Willing to support research in Germany (n = 48)	45 (92)
Give money	13 (27)
Fill out surveys	31 (63)
Provide patient data	31 (63)
Participate in practice-base research	15 (31)
Write or edit manuscripts	6 (12)
Other	2 (4)

*Values reported in frequencies (%) unless otherwise noted.

The value of research for different aspects of chiropractic practice was assessed using a Likert scale, anchored with the descriptors "extremely important" to "not at all important" (Table 4). Respondents considered research extremely important for the acceptance of chiropractic among other health care disciplines (65%); for scientific collaboration (51%); and, for the acceptance among patients (45%).

**Table 4:** Value of Research (n = 49)*

	Extremely important 1	2	3	4	Not at all important 5
**Improving practice**	19 (38.8)	17 (34.7)	10 (20.4)	3 (6.1)	-
**Acceptance (patients) **(n = 48)	22 (44.9)	8 (16.3)	12 (24.5)	5 (10.2)	1 (2.0)
**Acceptance (health care disciplines)**	32 (65.3)	10 (20.4)	5 (10.2)	-	2 (4.1)
**Acceptance (3^rd ^party payers) **(n = 48)	18 (36.7)	16 (32.7)	6 (12.2)	3 (6.1)	5 (10.2)
**Practice Guidelines**	15 (30.6)	15 (30.6)	14 (28.6)	1 (2.0)	4 (8.2)
**Scientific collaboration**	25 (51.0)	14 (28.6)	6 (12.2)	-	4 (8.2)

*Values reported in frequencies (%).

The last three items of the questionnaire consisted of open-ended questions. Not every participant responded to every question. All responses were recorded in Tables 5, 6, and 7, respectively. The question "In your opinion, what should be done to increase research efforts by the profession in Europe, and specifically in Germany?" elicited answers that can be categorized into acceptance (9), collaboration (8), research priorities (8), comparison (5), and publications (5) (Table 5). Acceptance of the chiropractic profession by the larger public and other health care professions was a primary concern, which in the opinion of some participants should be established before time and money is invested in research. Collaboration with universities, medical researchers and scientists as well as more intra-professional collaboration to conduct chiropractic research was thought to be important by eight of the participants. Several practitioners underlined the importance of comparing chiropractic to other manual methods or standard medical care to distinguish what chiropractors do. Increasing the amount of German research publications was thought to be important as well, either as a German journal or German translations of important research, to educate the public as well as other health care professions, lawyers, and others. Research priorities were mainly focused on musculoskeletal conditions.

**Table 5:** Improvement of research efforts*

**Acceptance**
• Acceptance of chiropractic in the health system (Germany only)
• Carefully position ourselves into a position of demand before jumping into Ins.
• 3rd party payers
• Educating the public about chiropractic
• First, chiropractic in Germany needs to be recognized before resources (time, effort, money) can be allocated to research.
• First, chiropractic should be a protected profession before we put effort in research because the results will be mixed with therapists who do chiropractic but are not allowed to.
• Start changing minds through information: "health comes from within" needs to be reinforced
• More practitioners in Germany (too few)
• Organize the profession

**Collaboration**
• A coordinated center with true PhD researchers working with Chiropractors
• Establish a European/German researching body/organization.
• Get Universities involved
• Set up a chiropractic school working with medical researchers
• Support of scientists by the national associations
• Work close together with other practitioners outside & within our practices to further our knowledge
• Greater exchange of information and experience between DC's
• Raising an obligatory contribution by national associations of their individual members together with the membership dues

**Research**
• Evaluating chiropractic care for musculoskeletal problems
• Evaluating chiropractic care in general
• More manuscripts published by German chiropractors for specific musculoskeletal conditions
• Ongoing access (only for chiros) by website as to the progress of projects.
• Suggest input parameters before projects from field doctors.
• Teach research methodology in a seminar
• The colleges must educate students to do research – it takes people who are interested and capable to conduct meaningful research
• More money

**Comparison**
• Comparison between Chiropractic Care & Medical care for Musculoskeletal conditions
• DC successes in comparison to other health professions in general
• Distance ourselves from resembling what medicine does
• Distinguish ourselves for where our best contribution is (a Chiropractic Adjustment)
• To show the difference in treatments between Chiropractors vs. Manual Therapists vs. Osteopaths vs. Physios

**Publications**
• German Journals
• Have studies translated in to German
• More manuscripts published by German chiropractors for specific musculoskeletal conditions
• More publishings in German magazines, etc. of efficacy of chiropractic care for musculoskeletal conditions
• Talk with other chiropractors about experiences and put them together in a paper

**Other comments**
• Form a school
• Information
• Support
• Think as a profession and not as individuals
• I do not think that research should be a priority in Germany at this point
• Don't know
• n/a
• ?

*Survey Question 41: In your opinion, what should be done to increase research efforts by the profession in Europe, and specifically in Germany?

**Table 6:** Most pressing issues in Germany*

**Recognition/Protection**
• A school and official licensure
• 3rd party payers
• Chiropractic licensing law
• Chiropractors must practice under Heilpraktiker license and compete with chiropraktikers (heilpraktikers) plus MD's who are allowed to practice "chirotherapy."
• Differentiate between other manipulative therapists
• Governmental recognition
• Laws providing protection/recognition
• Legislation – clear defined handbook
• Official acknowledgement of the profession
• Only chiropractors should be allowed to do chiropractic therapy
• Recognition
• Recognition from other health care professions
• To be accepted as a profession other than heilpraktikers
• To protect the name/title of chiropractor
• To establish it as its own profession and to regulate chiropractic
• Legal recognition

**Education**
• Chiropractic being taught in seminars to non-chiro students
• Professional education

**Publicity**
• Bad press
• Demand by the needing public – education and not jumping emotionally toward medically "acceptable" manipulative therapy
• Identity
• Patient Education.
• Public information/education about the profession
• Raise awareness

**Research**
• A coordinated center with true PhD researchers working with Chiropractors.
• Evaluating Chiropractic care in general
• Show that multiple adjustments are not creating hyper-mobility
• Side effects of chiropractic treatment efficacy
• Strokes

*Survey Question 42: In your opinion, what is currently the most pressing issue for the chiropractic profession in Germany?

**Table 7:** Other comments about research and the survey*

**Research in general**
• Do not know much about the "ins" and "outs" of research -> this is a great start though, we need to know the facts to support the success we get...and then get that out there. Unfortunately misinformed/false/not true medical and pharmaceutical information obstructs us. We do not want to get on the level of the groups that say we cause strokes...are no good etc. -> but we should get statistics on what they do/cause/kill...
• I'm not a researcher but why does it seem that serious flaws are found after studies are made?
• Research experience limited to "Diplom Arbeit."> Research in Germany from Germans about German chiros is what we need.
• Research is the basis of our profession. However, it needs to be made more public both inside and outside the practicing profession. Remember the passion and charisma with which B.J. presented his work?!
• Research seems to be most effective if done by educational institutions and national associations provided they have the basis. Your survey is O.K.
• Research world wide has an impact on my way of seeing chiropractic and how to apply it. My feelings however is that it has not reached other colleagues the same way – especially talking about SOT, AK, techniques; they have been left to the medial field and physiotherapists to be picked up and being sold to patients as their own discovery. "Very few" – if any – attend seminars in England
• Very little experience with research
• We desperately need scientific studies translated into German to use them in communication with MD's, patients, lawyers, etc.

**Chiropractic**
• Chiropractic as means to unite body, soul, and mind – to achieve equilibrium/Homeostasis – Happy People!
• German population needs to be educated on difference between Chiropractors (with a chiropractic education) and chiropraktikers, who learn technique only through weekend seminars.
• It is important that positive research is published in magazines and newspapers – there is too much negative publicity in Germany about Chiropractic
• Via sound and logical reasoning we should desire for the public to crave the answers to questions like "Why did I get sick?" rather than "What should I take – what shortage do I have." People should ask "How does health become lost" and "How can I learn to understand rather than not think at all more clearly?
• Clinical and scientific research is crucial for the future of chiropractic.

**Survey Comments**
• Average age of patient/social background/educational level would be interesting to know.
• Good survey – to obtain a summary of all survey's would be good
• Nice survey – Good luck with the stats!
• Question 38 was very difficult to answer
• Seems relevant, some scientists waste a lot of time and money. Observing Denmark research seems politically important.
• Survey is in my opinion too detailed. Subjects may tend to not take their time for answering correctly. Good professional layout and conduct.
• Too many questions
• Very thorough – good luck with it

*Survey Question 43: Please provide any other comments about your experience with research and about your impressions of this survey.

The most pressing issue for the chiropractic profession in Germany raised by the respondents was clearly the lack of recognition and licensure (Table 6). Issues in this category included a licensing law, differentiation from other manual practitioners/heilpraktikers, recognition by other health care professions and protection of the title "chiropractor." Publicity, research, and education were three other categories important to respondents.

Participants were also asked to provide any other comments about their experience with research. Three themes emerged: research in general, chiropractic in general, and comments about the survey. (Table 7) Several respondents commented that their experience with research was limited, but that research is important for the profession. The importance of making chiropractic research more public was stressed, within the chiropractic profession as well as with the general public and other health care professions.

## Discussion

The results of this survey suggest that this sample of chiropractors, most of whom practice in Germany, consider research important and are willing to support research. However, at this point in time, the priorities for most practitioners is to gain acceptance among patients and other health care professionals, establish professional licensure laws and protect the title "chiropractor."

The demographic characteristics of chiropractors who responded to this survey are very similar to those found in a survey done in The Netherlands in 2002. [11] The mean age of chiropractors in our survey was similar to that of Dutch chiropractors (mean age 38, SD 9.3). This has not changed since the last survey in Germany in 1997. However, if respondents to our survey are representative of German chiropractors, the proportion of female practitioners in Germany has grown from 23% to 37% in the past decade. In The Netherlands, approximately 32% of chiropractors are female. In a 2000 survey in the United Kingdom, 46% of the practitioners were female, whereas in a 1976 survey by Breen only 8% of the respondents were female. [12] There appears to be a shift in chiropractic education; ten years ago, most chiropractors who responded to a survey of chiropractic practice in Germany were trained in American schools (30% at Palmer College). [9] Our results show slightly more respondents graduated from AECC (35%). This trend is confirmed with the current student population: there is one German student currently enrolled at Palmer College, and nine of the 12 GCA student members are studying in the United Kingdom. (personal communication) In the United Kingdom, about 25% of chiropractors were trained within the country in the 1970's; in 2000, 82% reported to have completed their training in the UK. [12] A high proportion of non-Dutch practitioners practice in The Netherlands (43%)[11] and from our survey, 41% of non-German national respondents practice in Germany (Table 1). A likely explanation is the lack of chiropractic schools in continental Europe and thus a small number of native chiropractors in both countries.

Even though the importance of research is recognized, an average of only three hours per week is reportedly spent reading scientific literature by the respondents. The median of one hour is likely to be closer to the time the average practitioner spends reading. Few of the publications respondents read are peer-reviewed and indexed; most are newsletters of chiropractic organizations or free publications. Several respondents commented on the limited exposure to research, and some suggested offering a seminar on research methodology. This could be an important first step if measures to implement research activities in Germany are taken.

Compared to a sample of 1,245 American chiropractors in 1997 [14], respondents in our survey rate the value of research in different ways. (Table 4) Sixty-five percent of respondents to our survey view research as extremely important for the acceptance among other health care disciplines compared to 44% in the US survey. On the other hand, 45% of our survey respondents view research as extremely important for the acceptance among patients, compared to 51% of respondents in the US a decade ago. These data support the current priority of the chiropractic profession in Germany to establish acceptance among other health care practitioners and to gain licensure.

Two opposing opinions emerged from the responding chiropractors in Germany related to research activities: on the one hand, many practitioners suggest that more research should be published in German and by chiropractors in Germany, but on the other hand several practitioners think that money and effort should be put into gaining recognition within the German health care system first. (Tables 5 and 6) With such a small group of chiropractors in Germany and limited resources, it is a difficult task to gain both professional recognition and increase research activities. It appears that educating practitioners about resources such as open-access journals or information available through the ECU Website could help chiropractors gain access to research; these available resources in turn could potentially be used to educate the public as well as legislators about chiropractic.

### Limitations

The sample for the current survey included only members of the GCA and seminar participants (who were not necessarily GCA members). We did not attempt to find and include DC's who are not members of the association. However, it is estimated there are about 15 chiropractors practicing in Germany who are not GCA members. (personal communication)

Although the opportunity and interest to survey GCA members was fortuitous, a major limitation of this project and lesson learned, was that we did not allow enough time to develop the survey items. It became clear in the analysis phase, that we failed to clearly delineate the way in which we captured information about practice location, nationality, and GCA membership (i.e., some seminar attendees were not members of the GCA). From items 3, 8, 9, and 13 [see Additional file 1], we were able to show that three of 29 German nationals and one of 20 non-Germans practice outside of Germany and presumably never practiced in Germany. There were missing data for two non-German respondents. Because of the small survey sample and because we did not know if 4, 5 or 6 of respondents actually practiced outside of Germany, we chose to describe results from all respondents in this report. Therefore, our results should be interpreted with caution.

## Conclusion

The results of this survey indicate that currently the first priority for chiropractors who responded to this survey is gaining licensure for chiropractors in Germany. In addition, there is general interest to support and participate in research activities. As pointed out by some respondents, implementing research activities in chiropractic also has the potential to increase acceptance and establish chiropractic licensure. Practitioners voiced an interest in translations of key studies into German, which could be an important tool for educating legislative bodies as well as the public. An important next step may be to educate chiropractors in Germany about the resources available to read and interpret the scientific literature.

## Competing interests

The author(s) declare that they have no competing interests.

## Authors' contributions

IS conceived the idea for the study. IS and MAH contributed to the design and planning of the project. IS administered the survey to 37 participants at a seminar in Germany and prepared the mailings for 31 potential participants. MAH managed incoming data from the mail surveys. IS analyzed all data and prepared the first draft of the manuscript. MAH provided critical revisions for intellectual content. Both authors edited and approved the final version of the manuscript.

## Supplementary Material

Additional file 1 Appendix 1. Questionnaire Click here for file
