# Supplementary material for: A survey of chiropractors practicing in Germany: practice characteristics, professional reading habits, and attitudes and perceptions toward research
Source: Chiropr Osteopat. 2007 May 4;15:6. doi: 10.1186/1746-1340-15-6 (PMC1887523; doi:10.1186/1746-1340-15-6)
Supplement: Additional file 1 — Appendix 1. Questionnaire [file 1746-1340-15-6-S1.doc]

**Appendix 1. Questionnaire.**

# *Please check the one best response choice except where instructed otherwise.*

1. What is your gender? _1_❑ Female _2_❑ Male

2. In what year were you born? 19_____

3. What is your nationality? _1_❑ German _2_❑ Other _________________________

4. Where did you attend chiropractic school?

5. In what year did you complete this degree program? _______

6. Please list all of the degrees (incl. diplomates) you hold other than your chiropractic degree:

Degree Institution Year

7. How many years have you been practicing as a chiropractor? ____ years

8. How many years have you been practicing as a chiropractor in Germany? ____ years

9. How many years have you been practicing at your present location? ____ years

10. With what type of practice were you affiliated immediately after graduating? ***(Check all that apply)***

_1_❑ Associate/Employee _4_❑ Chiropractic group practice

_2_❑ Solo practice _5_❑ Multi-specialty group practice

_3_❑ GEP (Graduation Education Programme) _6_❑ Other _________________________

11. With what type of practice are you now affiliated? ***(Check all that apply)***

_1_❑ Associate/Employee _4_❑ Chiropractic group practice

_2_❑ Solo practice _5_❑ Multi-specialty group practice

_3_❑ GEP (Graduation Education Programme) _6_❑ Other _________________________

12. In how many clinics or practices do you currently see patients? ___ clinic(s)

13. Are any of your practice locations outside of Germany?

_1_❑ No _2_❑ Yes ***(Please specify)***____________________________________________

14. Please describe the setting of your practice location(s) in a general way (e.g. large city, rural, etc.)

15. Do you conduct activities to promote your practice?

_1_❑ No _2_❑ Yes ***(Please specify)***_____________________________________________

16. How many hours per week do you practice?

_1_❑ Less than 10 hours per week _4_❑ 31-40 hours per week

_2_❑ 10-20 hours per week _5_❑ More than 40 hours per week

_3_❑ 21-30 hours per week

1. Approximately how many hours a week do you spend on paperwork related to

your clinical practice? ____ hrs/week

18. Approximately how many hours a week do you spend on direct patient care? ____ hrs/week

19. Thinking about the past year, what is the average number of patients you see

in a week? ____ pts/week

20. Approximately what percentage of your patients are 6 years of age or younger? ____%

21. Approximately what percentage of your patients are 65 years of age or older? ____%

22. Approximately what percentage of your patients are female? ____%

23. In general, when a new patient calls your office to schedule their first appointment, what is the earliest appointment available according to your schedule?

Within ____ days

24. Please list the chiropractic techniques or systems you use in your office. List in order, starting with the technique you use **most often**.

25. Please list any other interventions you use in your office, including approaches like home exercise, nutrition counselling, and patient education:

26. What are your sources for new patients? Please provide information about the frequency you get referrals from specific sources as well (e.g. 1-2x per week, once a month, etc.).

27. What are the three most common reasons patients seek care in your office (e.g. the three most common chief complaints)? Please list in order with the most common complaint first.

1.

2.

3.

28. What are the three most common non-musculoskeletal complaints that patients seek care for in your office? Please list in order with the most common complaint first.

1.

2.

3.

29. Do you read journals related to chiropractic or other health care disciplines?

_1_❑ Yes _2_❑ No ***(SKIP to Q. 33)***

30. Approximately how many hours a week do you spend reading about chiropractic or other health care disciplines?

____ hours/week

31. Why do you usually read chiropractic or other scientific journals? ***(Check all that apply)***

_1_❑ Patient education _3_❑ Specific patient condition

_2_❑ Special interest area _4_❑ Other ________________________________

32. Which scientific journals do you read regularly? Please list paper and electronic publications.

33. Which electronic databases do you use to search for scientific literature? ***(Check all that apply)***

_1_❑ EMBASE _4_❑ MEDLINE (PubMed)

_2_❑ Index to Chiropractic Literature (ICL) _5_❑ None of the above

_3_❑ MANTIS _6_❑ Other ***(Please specify)***_________________

34. Please list any other types of electronic resources you use to obtain information relevant to your practice (e.g. listserves, websites, etc).

35. On average, how often do you use electronic resources to search for scientific literature?

____ time(s)/month

36. Have you ever published an article in a scientific journal?

_1_❑ No _2_❑ Yes

***If Yes****, please specify journal and year:*

37. Please indicate your personal view of the role and value of chiropractic research to each of the following.

Extremely Not at all

Important Important

Improving everyday

clinical practice 1 2 3 4 5

Acceptance of chiropractic

among patients 1 2 3 4 5

Acceptance of chiropractic

among other health care disciplines 1 2 3 4 5

Acceptance to 3^rd^ party payors 1 2 3 4 5

Helping establish practice

parameters/guidelines 1 2 3 4 5

Increasing collaboration & integration

of chiropractic within scientific circles 1 2 3 4 5

38. If the chiropractic profession were able to focus its research efforts on a single goal for the next five years, how would you rank the following in terms of their importance? ***(Please rank the items with 1-8, using 1 for the most important and 8 for the least important goal.)***

___ Improving the effectiveness of chiropractic education.

___ Evaluating the efficacy of chiropractic care for various musculoskeletal conditions.

___ Building the ability of the profession to conduct its own research.

___ Evaluating the validity and reliability of chiropractic techniques, procedures, and equipment.

___ Basic science research on the nature of the subluxation complex, and/or the physiological effects of adjustments.

___ Evaluating the efficacy of chiropractic care for various “visceral” conditions.

___ Health services research on chiropractic in the health industry (e.g. cost-effectiveness studies).

___ Other *(please specify)* __________________________________________________

39. Are you willing to support research efforts in Germany?

_1_❑ Yes _1_❑ No ***(SKIP to Q. 41)***

40. In what ways are you willing to support research efforts in Germany? ***(Check all that apply)***

_1_❑ Contribute money _4_❑ Participate in a practice-based research network

_2_❑ Complete surveys _5_❑ Edit/write manuscripts

_3_❑ Provide patient data _6_❑ Other ***(Please specify)*** _____________________

41. In your opinion, what should be done to increase research efforts by the chiropractic profession in Europe, and specifically in Germany? Please be specific.

42. In your opinion, what is currently the most pressing issue for the chiropractic profession in Germany? Please be specific.

43. In the space below, please provide any other comments about your experience with research and about your impressions of this survey. Your comments will be considered carefully as an important part of the evaluation.

***Please make sure that you have answered all the questions that apply to you.***

***Then, please return the survey to the person who administered it at the conference.***

***Thank you very much for your contribution!***
